# Crossing a CRISPR/Cas9 transgenic tomato plant with a wild-type plant yields diverse mutations in the F_1_ progeny

**DOI:** 10.3389/fpls.2024.1447773

**Published:** 2024-08-07

**Authors:** Yasuhiro Ito

**Affiliations:** Institute of Food Research, National Agriculture and Food Research Organization (NARO), Tsukuba, Ibaraki, Japan

**Keywords:** CRISPR (clustered regularly interspaced short palindromic repeat)/Cas9 (CRISPR associated protein 9)-mediated genome editing, tomato, RIN (ripening inhibitor), mutagenesis, GGPS2

## Abstract

Generating CRISPR/Cas9-mediated mutants in tomato (*Solanum lycopersicum* L.) involves screening shoots regenerated from cultured cells transformed with a T-DNA harboring sequences encoding Cas9 and single guide RNAs (sgRNAs). Production of transformants can be inconsistent and obtaining transformants in large numbers may be difficult, resulting in a limited variety of mutations. Here, I report a method for generating various types of mutations from one transgenic plant harboring the CRISPR/Cas9 system. In this method, a wild-type plant was crossed with a T_0_ biallelic mutant expressing two sgRNAs targeting the *RIPENING INHIBITOR* (*RIN*) gene, and the resulting F_1_ seedlings were classified using a kanamycin resistance marker on the T-DNA. Genotyping of the *RIN* locus revealed that kanamycin-sensitive F_1_ seedlings, which carried no T-DNA, always harbored the wild-type allele and a mutant allele from the transgenic parent. Kanamycin-resistant F_1_ seedlings, which do carry the T-DNA, harbored a variety of novel mutant alleles, but not the wild-type allele, suggesting that it was mutated during crossing. The novel mutations included one-base insertions or short deletions at each target site, or large deletions across the two target sites. This method was also successfully applied to produce mutations in *Geranylgeranyl pyrophosphate synthase 2* (*GGPS2*). Because this method involves crossing rather than transformation, it can be readily scaled up to produce numerous novel mutations, even in plant species or cultivars for which transformation is inefficient. Therefore, when initial transgene experiments fail to induce the desired mutation, this method provides additional opportunities for generating mutants.

## 1 Introduction

The CRISPR/Cas9 system produces mutations at a target site in the genome; although the target requires an NGG protospacer-adjacent motif (PAM), it can be altered flexibly by customizing 20-nt sequences in a single guide RNA (sgRNA) (Jinek et al., 2012). The Cas9 nuclease forms a complex with the sgRNA and cleaves double-stranded DNA at the site determined by the sgRNA. The cleaved site is repaired via error-prone non-homologous end-joining (NHEJ), resulting in mutations at the target site. One-base insertions or deletions of a few to more than several hundred bases are observed frequently, while base substitutions occur infrequently (Zhang et al., 2014; Ito et al., 2015). However, there appear to be no obvious rules for repairing the double-stranded DNA break at the target site. Among mutations that modify gene function, frameshift mutations producing a knockout gene or a gene encoding a C-terminal truncated protein are readily expected. For example, C-terminal truncation of a tomato (*Solanum lycopersicum* L.) glutamate decarboxylase mediated by genome editing removed a domain regulating enzyme activity, resulting in constitutive enzyme activity and hyper-accumulation of γ-aminobutyric acid (GABA) (Nonaka et al., 2017). By contrast, when a specific mutation is desired, such as an in-frame deletion mutation to remove one or several amino acids from a protein or a large deletion mutation, researcher may have to screen many mutants originating from multiple mutagenesis experiments.

To activate the CRISPR/Cas9 system in most plants, a vector that includes a T-DNA encoding sequences for Cas9 and sgRNA is constructed; the T-DNA is then inserted into the genome by Agrobacterium (*Agrobacterium tumefaciens*)–mediated infection. Except for *Arabidopsis* (*Arabidopsis thaliana*), production of transgenic plants typically requires tissue culture. Transformation and regeneration protocols have not yet been established for all species, and many are efficient only for a specific cultivar. Using tissue culture to regenerate shoots from cells infected with Agrobacterium is a bottleneck to producing mutants. This can be a limiting factor in generating a variety of mutations through genome editing.

In this study, I demonstrated that various novel mutations can be produced in F_1_ progeny by crossing a wild-type tomato plant with a transgenic line expressing transgenes for the CRISPR/Cas9 system. In fertilized cells, a wild-type allele from pollen can be targeted by Cas9 derived from the genome of the transgenic line just after fertilization or at an early developmental stage. This method can be used to increase the variety of mutations created, even when only a limited number of mutations are identified among the first generation of transgenic plants.

## 2 Materials and methods

### 2.1 Plant transformation using the CRISPR/Cas9 system expressing plasmid vectors

The CRISPR-P program (http://crispr.hzau.edu.cn/CRISPR2/) (Lei et al., 2014) was used to select targeting sites in the *RIPENING INHIBITOR* (*RIN*) and *Geranylgeranyl pyrophosphate synthase 2* (*GGPS2*) genes. Plasmid vectors expressing *Cas9*, *NptII*, and two sgRNAs were constructed from pZK_gYSA_FFCas9 (Mikami et al., 2015). The plasmids were transformed into the wild-type tomato (*Solanum lycopersicum* L.) cultivar Ailsa Craig (AC) via the Agrobacterium*-*mediated method according to a procedure described previously (Sun et al., 2006). A T_0_ biallelic mutant was grown to the flowering stage, and the anthers were removed from the flowers at the pre-anthesis stage. The flowers were pollinated using pollen from AC flowers. Resulting F_1_ seeds were sterilized and placed on germination medium [1.5 g/L Hyponex powder (Hyponex Japan), 5 g/L sucrose, 8 g/L agar, pH 5.8] with 25 mg/L kanamycin. Germinated seedlings were classified into kanamycin-sensitive or -resistant groups according to root growth into the medium.

### 2.2 PCR amplification, heteroduplex mobility assay, and sequencing analysis

Genomic DNA was prepared from seedlings. A 3-mm-square leaf fragment was homogenized with 50 μL of extraction buffer [100 mM Tris-HCl (pH 9.5), 1 M KCl, 10 mM EDTA] in a 1.5-mL tube and then incubated at 95°C for 5 min. After centrifugation at 13,000 ×g for 3 min at 4°C in a microcentrifuge, 5 μL of supernatant was diluted with 45 μL of 1/10 TE buffer [1 mM Tris-HCl (pH 8), 0.1 mM EDTA] and subjected to PCR amplification. Target genomic regions were amplified using KOD FX neo polymerase (Toyobo). Primer sequences are listed in **Supplementary Table 1**. A heteroduplex mobility assay (HMA) was performed by electrophoresis of amplified DNA fragments in a 5% (w/v) acrylamide gel in 0.5× Tris-borate-EDTA buffer. For DNA sequencing, amplified samples were cleaned using a MagExtractor kit (Toyobo) and labeled using a BigDye Terminator v3.1 Cycle Sequencing kit (Applied Biosystems). DNA sequences were determined using a SeqStudio Genetic Analyzer (Applied Biosystems).

## 3 Results

**Figure 1:**
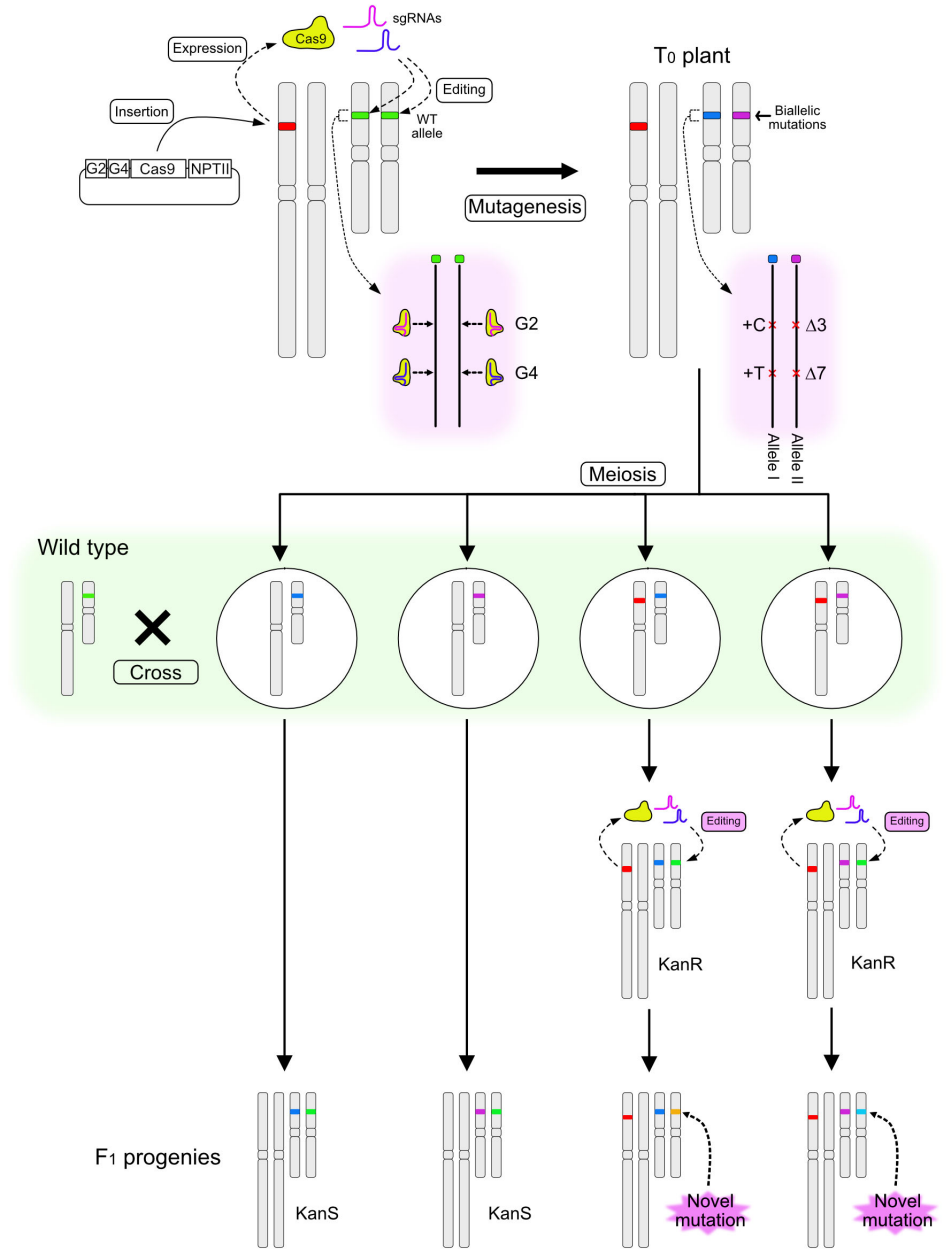
Schematic diagram illustrating a method for inducing novel mutations in the F_1_ generation. A T-DNA containing sequences encoding two sgRNAs (G2 and G4), Cas9, and NPTII is introduced into the tomato genome and induces mutations at the target locus. A biallelic T_0_ plant is pollinated using wild-type (WT) pollen. F_1_ progeny are selected on the basis of kanamycin sensitivity. Kanamycin-resistant (KanR) plants carry novel mutant alleles, which are generated from a WT allele immediately after fertilization or during early embryo development, whereas kanamycin-sensitive (KanS) progeny carry the intact WT allele.

**Figure 2:**
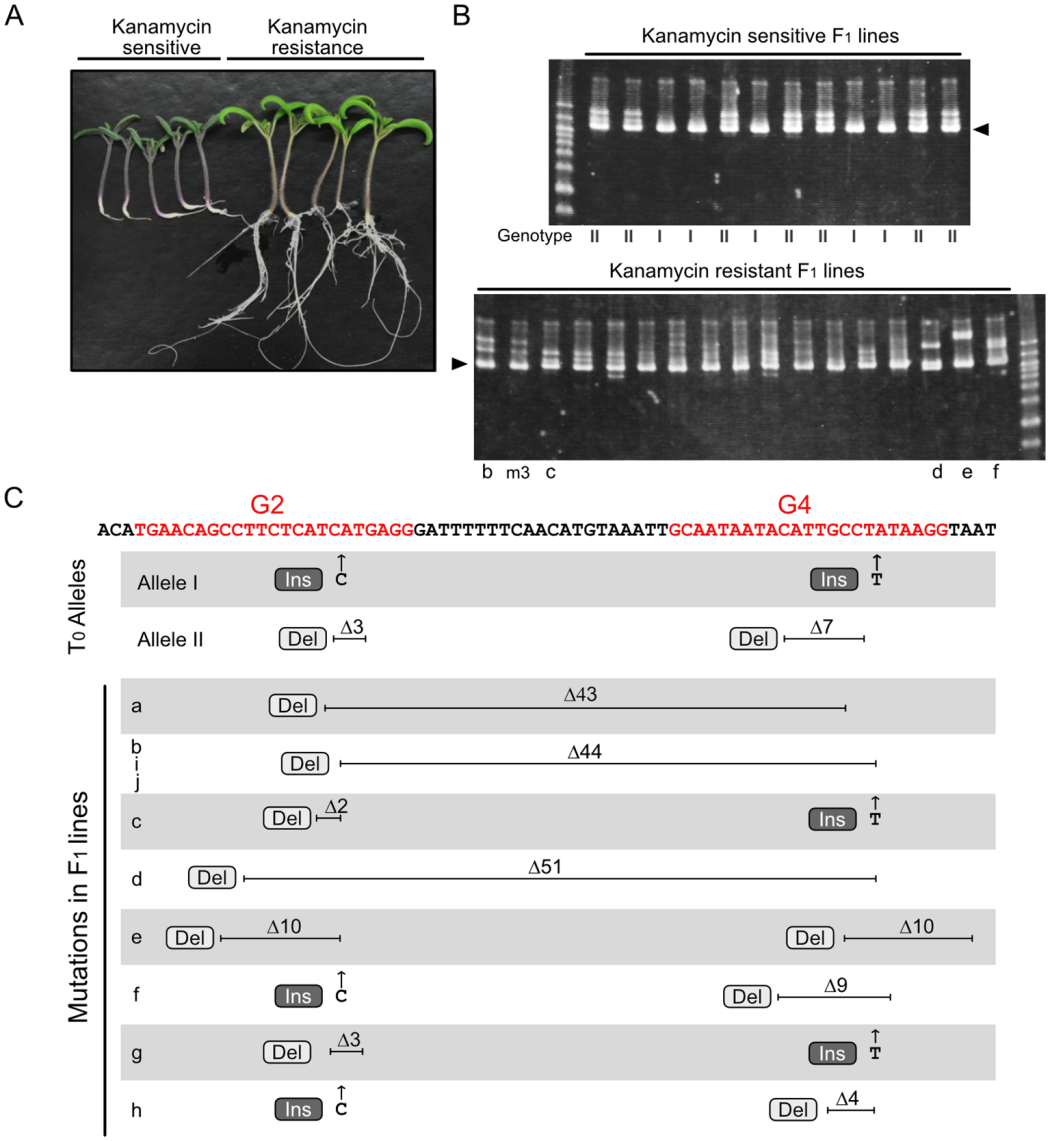
Novel mutations at the *RIN* locus identified in F_1_ progeny. **(A)** F_1_ seedlings grown on kanamycin-containing medium. **(B)** Heteroduplex mobility assay for the *RIN* gene fragment containing two sgRNA-targeted regions. Arrowheads show the common major bands, which are composed of homoduplexes. Other minor bands, which are presumably heteroduplexes composed of different combinations of diverse mutated fragments. Genotype I and II represent WT/Allele I and WT/Allele II, respectively; representative electropherograms of these genotypes are shown in **Supplementary Figure 1B**. Letters below each lane correspond to the lines indicated in panel **(C)** and **Supplementary Figure 1B**. This image shows representative results; additional electrophoresis images are shown in **Supplementary Figure 1A**. **(C)** Parental mutations and novel mutations identified in F_1_ progeny. Red letters in the sequence indicate the target regions of the two sgRNAs, G2 and G4, including the PAM. Electropherograms of the sequencing analysis are shown in **Supplementary Figure 1B**. Ins, insertion; Del, deletion.

The method for inducing novel mutations in F_1_ progeny after crossing a T_0_ mutant with a wild-type (WT) plant is illustrated in **Figure 1**. A binary vector designed to induce CRISPR/Cas9-mediated mutations at the *RIPENING INHIBITOR* (*RIN*; Solyc05g012020) locus in the tomato genome was constructed. *RIN* encodes a MADS-box transcription factor regulating tomato fruit ripening (Vrebalov et al., 2002; Ito et al., 2017), and several studies have targeted this locus for mutation using the CRISPR/Cas9 system (Ito et al., 2015; Li et al., 2020; Nizampatnam et al., 2023). The T-DNA was designed to express *Cas9*, two sgRNAs that target genomic regions within *RIN* located 20 bp apart, and *NptII* conferring kanamycin resistance. T_0_ plants were regenerated after Agrobacterium-mediated transformation of the tomato cultivar Ailsa Craig (AC), and a line with biallelic mutations at the *RIN* locus was identified. One allele had insertions of one cytosine base at the Guide 2 (G2)–targeted site and of one thymine at the Guide 4 (G4)–targeted site (+C/+T) (Allele I), and the other allele had a three-base deletion at the G2 site and a seven-base deletion at the G4 site (Δ3/Δ7) (Allele II) (**Figures 1**, **2C**). The T_0_ biallelic mutant was grown to the flowering stage. After meiosis, egg cells carrying one of the mutant alleles and with or without the T-DNA were produced; these were fertilized using pollen from AC harboring the WT allele (**Figure 1**). The F_1_ seeds were placed onto kanamycin-containing medium. Regardless of whether the seeds carried the T-DNA or not, germination was not affected by kanamycin, but root growth of the seedlings could be clearly classified as kanamycin resistant or kanamycin sensitive (**Figure 2A**), indicating segregation of the T-DNA among the progeny.

A fragment of the *RIN* locus encompassing the two target sites was amplified and analyzed using a heteroduplex mobility assay (HMA), which distinguishes different types of heteroduplexes formed from partially mismatched DNA fragments. Analysis of the kanamycin-sensitive lines revealed two electrophoresis patterns, most likely representing heteroduplexes of the WT allele and Allele I and of the WT allele and Allele II, respectively (**Figure 2B**, upper panel). By contrast, the HMA electrophoresis patterns for the kanamycin-resistant lines were diverse (**Figure 2B**, lower panel, **Supplementary Figure 1A**). In addition to a common major signal composed of homoduplexes with similar mobility, diverse minor signals, presumably comprising heteroduplexes between various mutated gene fragments, were observed in each sample, suggesting that different mutations might have been generated in each F_1_ line.

Sequencing of the amplified DNA fragments from F_1_ lines showing kanamycin sensitivity revealed only two types of electropherogram pattern. The electropherograms showed overlapping signals from two alleles, the WT allele and either Allele I or Allele II (**Supplementary Figure 1B**), indicating that the allele from WT pollen was intact. By contrast, the kanamycin-resistant F_1_ lines had diverse genotypes. Analyses of these F_1_ lines identified overlapping signals representing two or more different alleles (**Supplementary Figures 1B, C**). Analyses of 10 electropherograms showing two overlapping alleles revealed the presence of one parental mutant allele (Allele I or Allele II) and another novel mutant allele; however, the WT allele was not detected in any of the F_1_ lines. Among 10 novel mutant alleles, five had large deletions across the two sgRNA target regions, and the other five had point mutations in both target regions (**Figure 2C**, **Supplementary Figure 1B**). Of the five large deletion mutations, three were an identical 44-base deletion and the other two were located in a similar region, with both terminals of each deletion located within the targets of the two sgRNAs (**Figure 2C**, lines a, b, d, i, and j), suggesting that cleavage at the two target regions occurred synchronously. Because of the intricate patterns, genotypes could not be determined from electropherograms with three or more overlapping alleles (**Supplementary Figure 1C**). These electropherograms, however, consisted of one main signal and other multiple minor signals, and the main signals were either Allele I or Allele II but not the WT allele. This suggests that the alleles indicated by the minor signals were derived from the WT allele and were mutated independently after the first division of the fertilized cell (**Supplementary Figure 2B**). These results indicate that WT alleles derived from pollen are efficiently attacked by Cas9 during fertilization and early embryogenesis. Throughout the genotyping experiments, all F_1_ plants contained either Allele I or Allele II; no plants with genotypes that would have been generated from self-fertilization of the T_0_ mutant were observed. These results are consistent with the assumption that the T_0_ plant was a biallelic mutant and that outcrossing with WT was performed appropriately.

**Figure 3:**
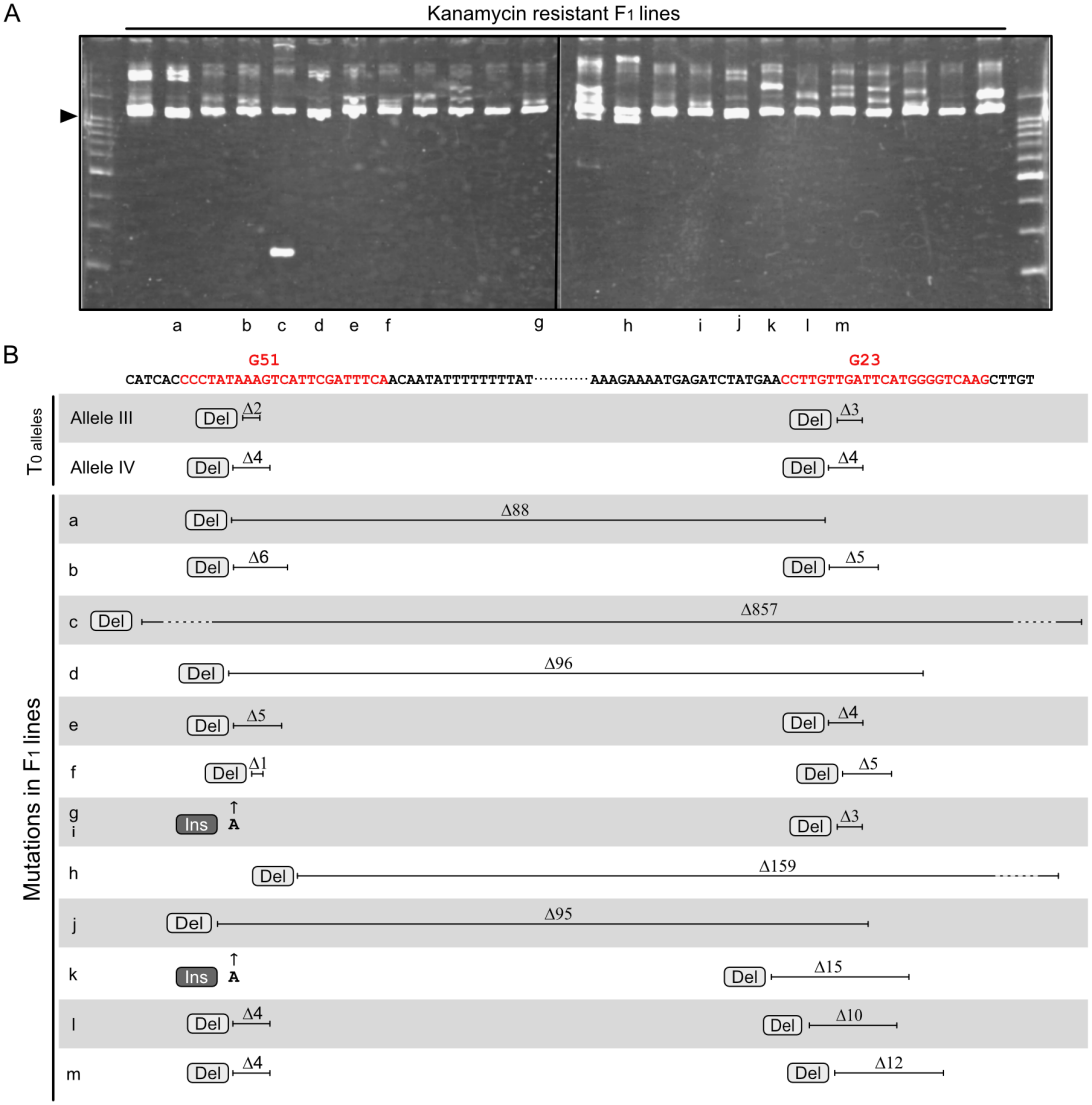
Novel mutations at the *GGPS2* locus identified in F_1_ progeny. **(A)** Heteroduplex mobility assay for the *GGPS2* gene fragment containing two sgRNA-targeted regions. Letters below each lane correspond to the lines indicated in panel **(B)** Arrowheads show the common major bands, which are composed of homoduplexes. Other minor bands, which are presumably heteroduplexes composed of different combinations of diverse mutated fragments. **(B)** Parental mutations and novel mutations identified in F_1_ progeny. Red letters in the sequence indicate target regions of the two sgRNAs, G51 and G23, including the PAM. Electropherograms of the sequencing analyses are shown in **Supplementary Figure 3**. Ins, insertion; Del, deletion.

To evaluate the suitability of this mutagenesis method for other loci, the *Geranylgeranyl pyrophosphate synthase 2* (*GGPS2*; Solyc04g079960) gene was targeted. *GGPS2* encodes an enzyme involved in the carotenoid biosynthesis pathway and is specifically upregulated during ripening (Fujisawa et al., 2013; Ito et al., 2020). As in the experiment targeting the *RIN* gene, two regions in the *GGPS2* gene located close together were chosen as targets for the two sgRNAs (**Figure 3B**). A vector containing a T-DNA expressing the two sgRNAs and *Cas9* was transformed into tomato cultivar AC, and a T_0_ plant with biallelic mutations at the *GGPS2* locus was selected as the parent for crossing. The T_0_ plant had two mutant alleles: one carrying a two-base deletion at the Guide 51 (G51)–targeted site and a three-base deletion at the Guide 23 (G23)–targeted site (Allele III), and the other carrying a four-base deletion at the G51-targeted site and a four-base deletion at the G23-targeted site (Allele IV) (**Figure 3B**). After fertilization with pollen of the WT cultivar AC, F_1_ seedlings showing kanamycin resistance were selected. HMA revealed diverse electrophoresis patterns for amplified fragments of the target region from F_1_ lines (**Figure 3A**), suggesting that independent mutagenesis events had occurred among these lines. DNA sequencing analysis of the amplified fragments allowed the genotypes of lines with two overlapping electropherograms, although not those with three or more overlapping electropherograms, to be determined, as in the experiment with *RIN*. Genotyping analysis of 13 F_1_ lines with two alleles revealed a novel mutant allele with either Allele III or Allele IV in each line (**Figure 3B** and **Supplementary Figure 3**). Of the novel mutant alleles, eight had point mutations at both target regions, and the other five had long deletions across the two target sites. Of the long-deletion alleles, two had extended deletions over the two target regions, which were 159 and 857 bp, respectively (**Figure 3B**, **Supplementary Figure 3C**).

## 4 Discussion

CRISPR/Cas9 is an excellent system for developing mutants at a target genomic region in many organisms, and diverse mutants have been developed in tomato using this approach (Larriba et al., 2024). The conventional method to produce mutants in tomato and many other plant species involves transforming Agrobacterium carrying a vector containing a T-DNA harboring the CRISPR/Cas9 system into a plant tissue, followed by *in vitro* tissue culture to regenerate transgenic shoots. In many plants, the procedure for regenerating shoots from transformed cells in tissue culture represents a critical bottleneck for obtaining large numbers of mutants. In addition, some kanamycin-sensitive tomato shoots regenerate under kanamycin selection, and such false-positive plants have no T-DNA and thus no mutation. Furthermore, regenerated tomato plants often have a tetraploid genome due to duplications arising during tissue culture (Sigareva et al., 2004). These factors reduce the efficiency with which mutants can be produced. In this study, I demonstrated that the F_1_ progeny of a cross between a tomato plant transformed using the CRISPR/Cas9 system and a WT plant harbor diverse, novel mutations not found in the parental mutant. The method for producing these mutants is simple and can be readily scaled up to generate many novel and diverse mutants; it just requires crossing a transformant possessing a T-DNA with a WT plant. Ideally, the desired mutant would be found among regenerated T_0_ plants. However, if candidate mutants are not identified among the regenerated plants, this method may provide another chance to obtain a variety of mutations, expanding the possibility of identifying mutants with the traits of interest. For example, an in-frame deletion mutation of codons encoding one or more amino acids, i.e., deletion of three bases or multiples of three bases, may be desired to remove amino acids at an active site or a phosphorylation site of a protein, which might change protein function, enzyme activity, or protein stability. In-frame deletion mutation of *RIN* changes the ripening phenotype of tomato (Nizampatnam et al., 2023). In this study, I found a mutant carrying a 51-bp deletion mutation at the *RIN* locus (**Figure 2C**), which resulted in an in-frame deletion of 17 amino acids. A long deletion may be useful for developing a protein with a defective domain or for removing the entire function of genes specifying long non-coding RNAs, which are transcripts of more than 200 nt that lack an evident open reading frame (Cao et al., 2024). Applying this method may be especially useful for other plant species or even tomato cultivars in which production of transgenic plants occurs with low efficiency. For example, there are well-established protocols for producing transgenic tomato plants, but the transformation efficiency varies between cultivars and production is not always stable between experiments (Sigareva et al., 2004; Sun et al., 2006; Rai et al., 2012). Even if only a few transgenic plants are obtained from such low-efficiency experiments, the method described here will increase the variety of mutations available in the next (F_1_) generation. In this study, T_0_ plants were crossed as the female parent with WT plants as the male parent, but reciprocal crosses were not performed. Crossing with a T_0_ plant as the male parent may also be effective for inducing novel mutations in the F_1_ progenies. F_1_ mutants obtained using this method carried biallelic (or multiallelic) mutations but were not homozygous mutants. To examine the effects of a mutation, a homozygous mutant should be selected among plants in the next (F_2_) generation.

To efficiently induce mutations using this method, the parental T_0_ plant requires sufficient activity of transgenes, e.g., sgRNA expression and Cas9 activity. T_0_ plants with biallelic mutations may be better candidates than plants carrying monoallelic mutations, which may have insufficient Cas9 activity to induce a mutation in another WT allele. However, monoallelic mutants carrying the transgene can produce novel mutations in their progeny by self-fertilization (Fauser et al., 2014). In addition, screening using kanamycin resistance is an effective way of selecting F_1_ plants with active transgenes and avoiding those with silenced transgenes. Prior to DNA sequencing, HMA electrophoresis patterns for a target genomic region are a good indicator of diverse mutations among F_1_ plants. Although the patterns may not be helpful for detecting heteroduplexes between fragments with only one base mismatched (Ito et al., 2015), a modified HMA that detects such slight mismatches has recently been developed (Kakui et al., 2021).

Sequencing analyses in this study revealed that F_1_ plants harboring the T-DNA possessed novel mutant alleles; plants with two alleles were frequently identified, and plants with more than three alleles were also generated. The two alleles comprised one allele from the mutant parent and one novel allele representing a mutated form of the WT allele, suggesting that the novel mutation was generated just after fertilization and before the first cell division of the fertilized cell (**Supplementary Figure 2A**). In F_1_ plants with more than three alleles, the mutagenesis would have occurred after the first cell division (**Supplementary Figure 2B**). Plants with two alleles are useful because genotyping from the sequencing electropherogram is easy and the genotypes of the progeny are predictable. The T-DNA that was introduced into the tomato genome in this study uses AtU6 and parsley ubiquitin promoters to induce the sgRNAs and *Cas9*, respectively (Mikami et al., 2015). Although these promoters are originally expected to drive during cell culture to induce mutations, the activity also appeared to be sufficient in fertilized cells.

## 5 Conclusion

This study demonstrated that CRISPR/Cas9-mediated mutagenesis occurs frequently within fertilized cells produced by crossing a WT tomato plant with a transgenic tomato plant harboring the CRISPR/Cas9 system. This simple crossing makes it easy to obtain F_1_ seeds with a wide variety of novel mutations at the target site. If a specific mutation is desired but not identified among T_0_ populations, subsequent crossing with a WT plant will provide another reliable chance for obtaining the desired mutations. This method offers promise for specific cultivars or plant species that only yield transgenic plants at a low efficiency.

## Data availability statement

The original contributions presented in the study are included in the article/**Supplementary Material**. Further inquiries can be directed to the corresponding author.

## Author contributions

YI: Writing – review & editing, Writing – original draft, Investigation, Funding acquisition, Conceptualization.
