# Supplementary material for: Crossing a CRISPR/Cas9 transgenic tomato plant with a wild-type plant yields diverse mutations in the F1 progeny
Source: Front Plant Sci. 2024 Aug 7;15:1447773. doi: 10.3389/fpls.2024.1447773 (PMC11335661; doi:10.3389/fpls.2024.1447773)
Supplement: Supplementary file 1 [file DataSheet_1.pdf]

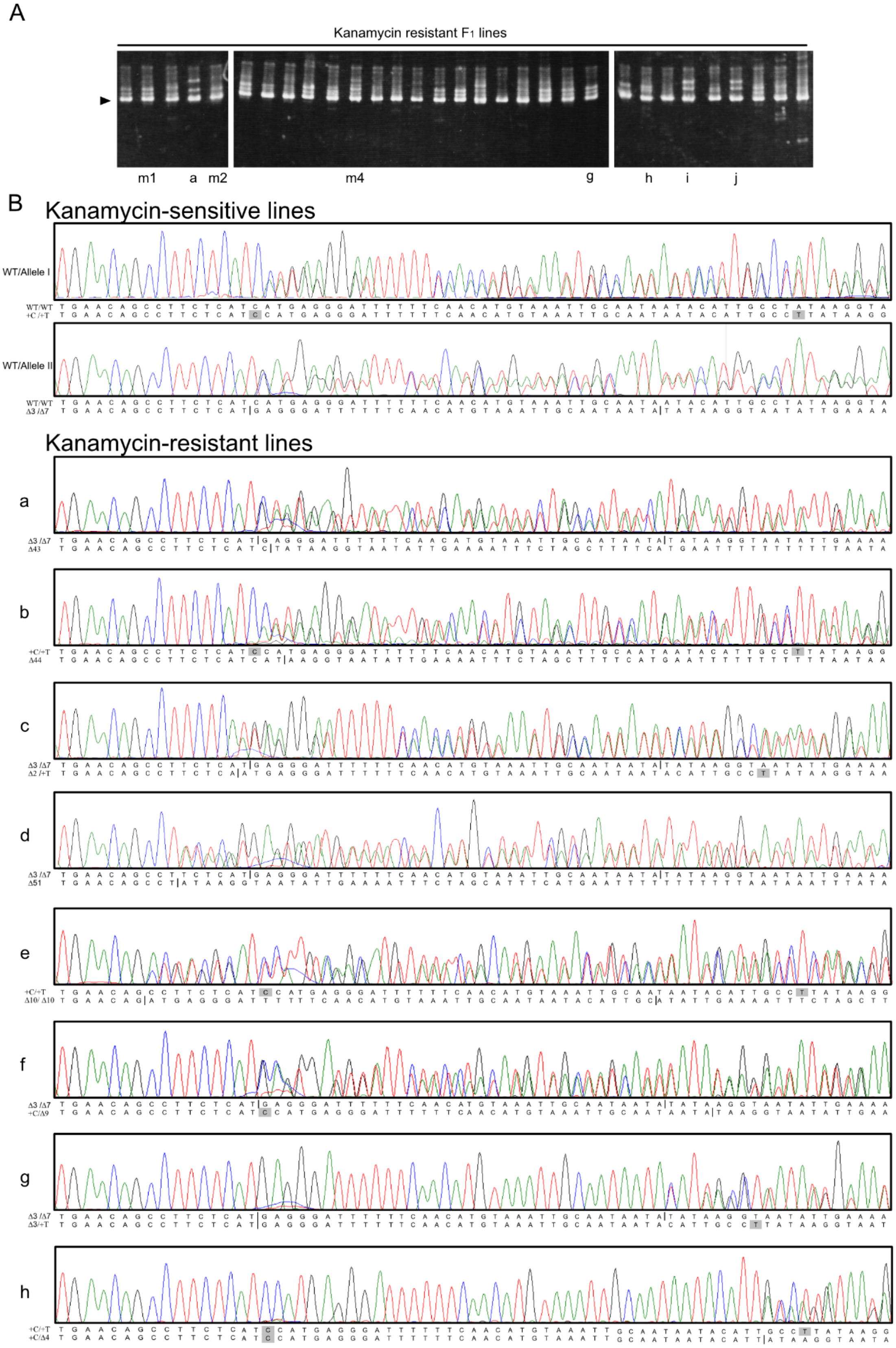
A
Kanamycin resistant F1 lines
m1
a
m2
m4
g
h
i
j
B
Kanamycin-sensitive lines
WT/Allele I
WT/WT
+C/+T
WT/Allele II
WT/WT
Δ3/Δ7
Kanamycin-resistant lines
a
Δ3/Δ7
Δ43
b
+C/+T
Δ44
c
Δ3/Δ7
Δ2/+T
d
Δ3/Δ7
Δ51
e
+C/+T
Δ10/Δ10
f
Δ3/Δ7
+C/Δ9
g
Δ3/Δ7
Δ3/+T
h
+C/+T
+C/Δ4

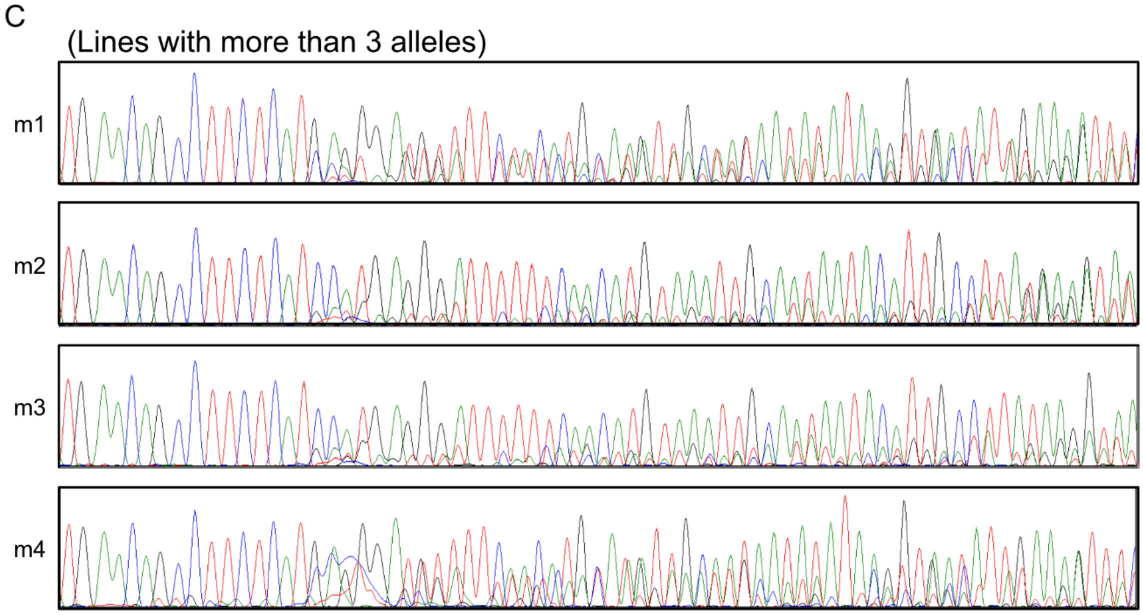

Supplementary Figure 1 | Mutations at the *RIN* locus detected by gel electrophoresis and DNA sequencing. (A) Heteroduplex mobility assay for the *RIN* gene fragment from F_1 lines derived from a cross between a wild-type (WT) parent and a T_0 parent harboring the T-DNA and biallelic mutations in *RIN*. Arrowhead shows the common major bands, which are composed of homoduplexes, and other minor bands, which are presumably heteroduplexes composed of different combinations of diverse mutated fragments. Letters below each lane correspond to the lines indicated in Figure 2C and panel B. These gel electrophoresis images and those in Figure 2B were obtained from a series of experiments. **(B)** Electropherograms of the mutation sites at the *RIN* locus. Electropherograms represent target regions of G2 and G4 (Figure 2C). All kanamycin-sensitive lines tested displayed one of the two electropherogram patterns shown, which comprise overlapping patterns for the WT allele and Allele I or the WT allele and Allele II. This suggests that the WT allele was intact after crossing. By contrast, the WT allele was not identified among kanamycin-resistant F_1 lines, but diverse electropherogram patterns were observed comprising two or more overlapping electropherogram patterns. The presence of two patterns allowed novel mutations to be identified; however, it was difficult to determine the sequence from three or more overlapping patterns (panel C). All of the electropherograms showed a main pattern representing either Allele I or Allele II, suggesting that the WT allele was mutated independently after division of the fertilized cell resulting in a chimeric plant (Supplementary Figure 2B). Lines among text sequences indicate deletion sites, and highlighted letters indicate base insertions. **(C)** Electropherograms of the mutation sites at the *RIN* locus with three or more alleles.

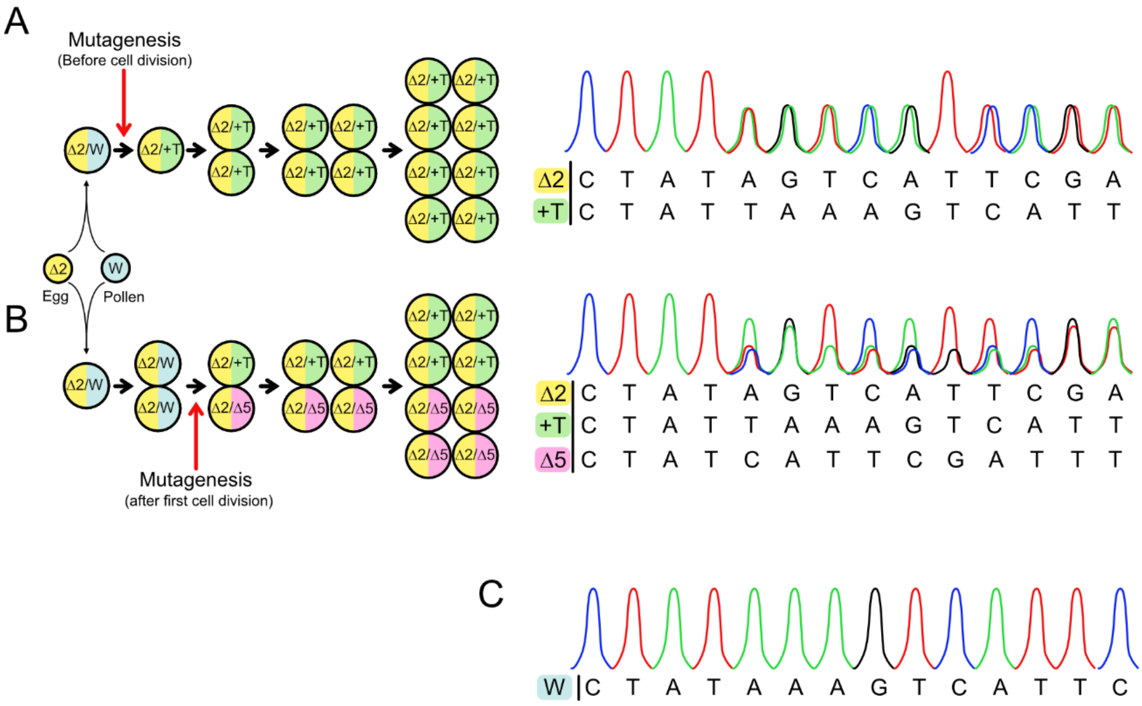

Supplementary Figure 2 | Schematic representation of allele compositions in F_1 lines.

This schematic represents an instance of the G51 target site in *GGPS2*. Pollen from a wild-type (W) plant is used to fertilize an egg cell with a 2-bp deletion allele (Δ2) from the mutant parent. (A) If mutagenesis of the wild-type allele occurs before the first cell division (a thymine insertion; +T), the plant is composed of cells with a single genotype (Δ2/+T), and an electropherogram analysis detects overlapping signals at a similar intensity. (B) If mutagenesis occurs after the first cell division, the two cells harbor independent genotypes (Δ2/+T and Δ2/Δ5) and the plant is a chimera. The electropherogram detects three overlapping signals: one main signal representing the parental allele (Δ2) and two minor signals corresponding to the induced mutant alleles (Δ5 and +T). (C) Electropherogram of the wild type.

A Guide 51 for GGPS2

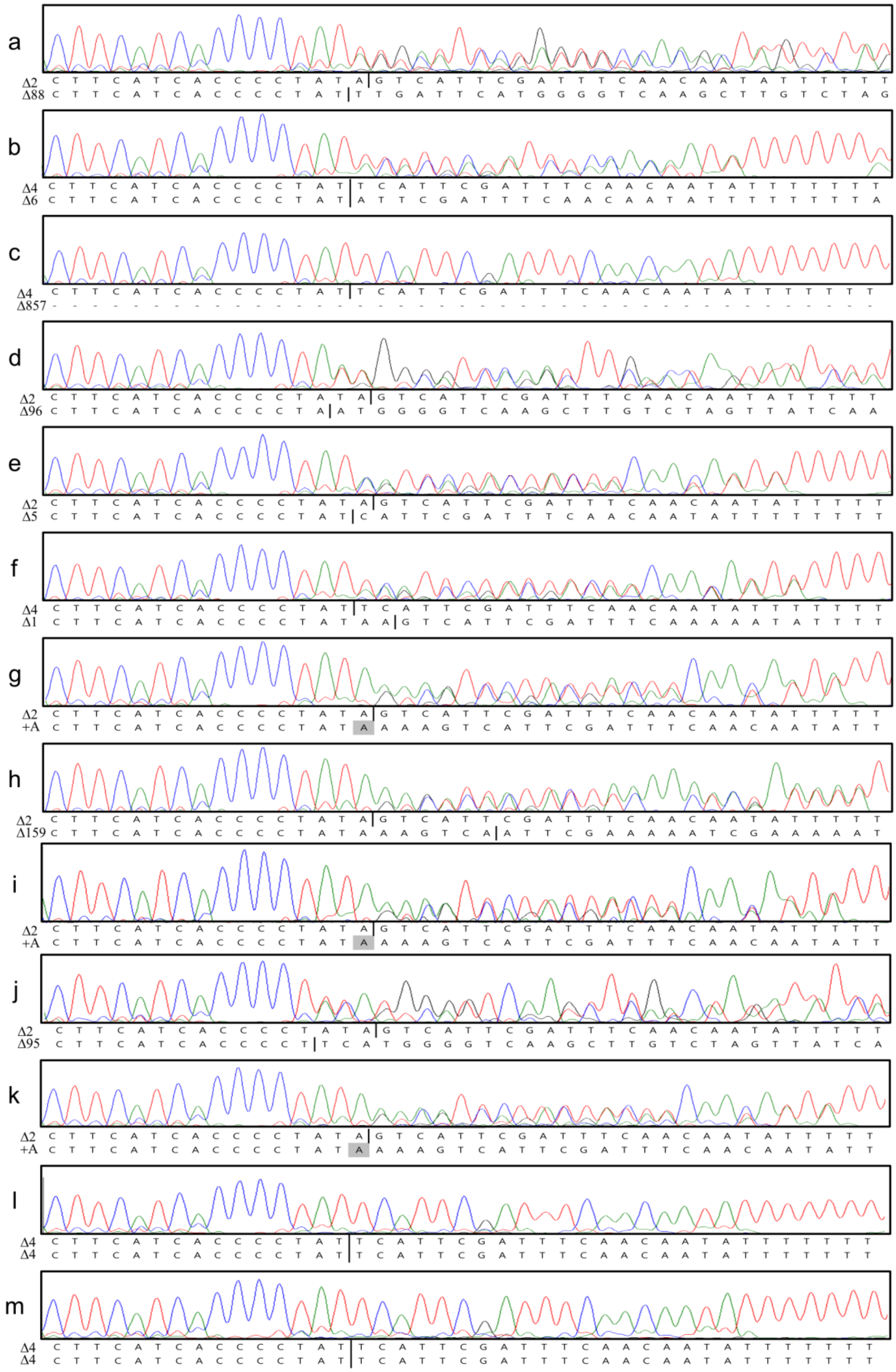

B Guide 23 for GGPS2

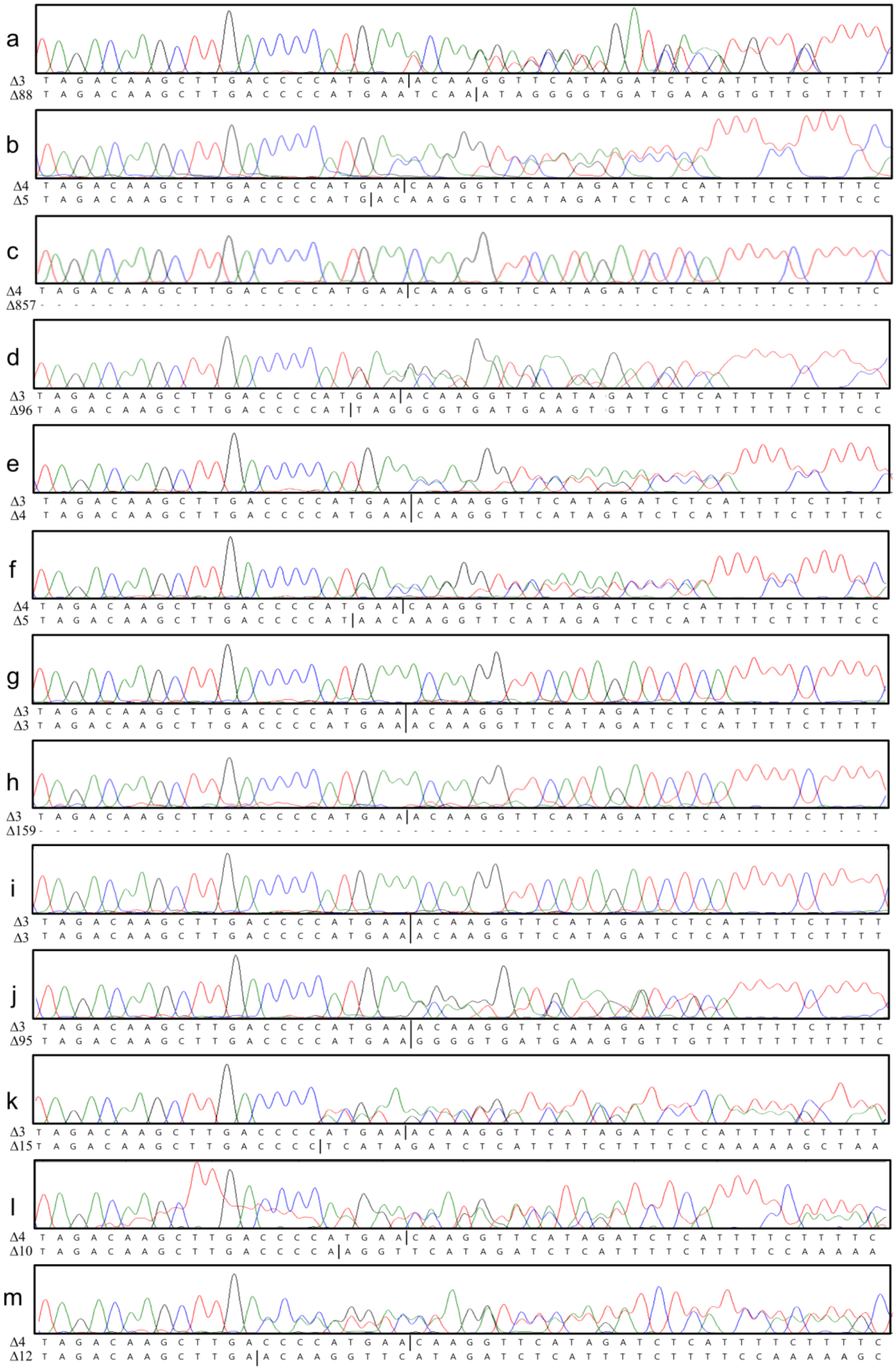

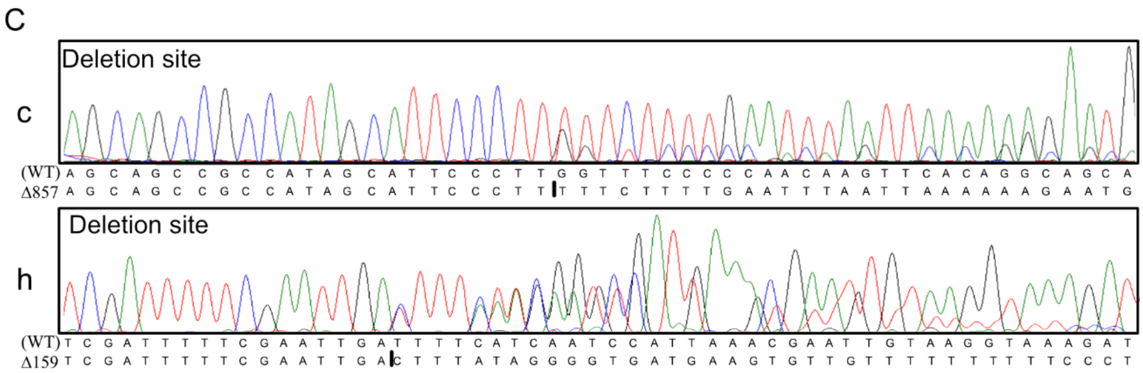

Supplementary Figure 3 | Electropherograms of the mutation sites at the *GGPS2* locus. Analysis of genomic DNA of F_1 lines derived from a cross between a wild-type (WT) parent and a T_0 parent harboring biallelic mutations in *GGPS2*. F_1 lines producing two overlapping electropherograms were analyzed. **(A)** Electropherograms of the G51 target region (Figure 3B). **(B)** Electropherograms of the G23 target region (Figure 3B). Sequencing was conducted in the reverse direction. **(C)** Electropherograms of the long deletions found in lines c and h. The indicated sites show the ends of the deletions. Lines among text sequences indicate deletion sites, and highlighted letters indicate base insertions.

Supplementary Table 1 Oligonucleotides used in this study

Name	Primer sequence (5′ to 3′)
RIN-F2	CACAACACATTCTTGATCAACT
RIN-R3	ACTCCAAATTCAAAGCATCCATCC
GGPS-F1	AAGAATTGTTTGGTTGACATATATG
GGPS-R1	CTTTATGATTCGTCGGCTTTCCACG
